# Culturable Airborne Microorganisms in Urban and Coastal Recreation Areas (Southern Baltic Sea)

**DOI:** 10.1007/s00248-026-02729-y

**Published:** 2026-03-26

**Authors:** Piotr Perliński, Zbigniew Jan Mudryk, Marta Zdanowicz, Łukasz Kubera

**Affiliations:** 1 Department of Experimental Biology, Institute of Biology, Pomerania University in Słupsk, Arciszewskiego 22B, 76-200 Słupsk, Poland; 2 Department of Microbiology and Immunobiology, Faculty of Biological Sciences, Kazimierz Wielki University, Al. Powstańców Wielkopolskich 10, 85-090 Bydgoszcz, Poland

**Keywords:** Baltic Sea, Bioaerosols, Coastal areas, Urban areas, Airborne microorganisms

## Abstract

Microbiological studies of bioaerosols werecarried out in urban and coastal recreation areas in a marine town located in the southern Baltic Sea region. The present study showed that the most abundant culturable airborne microorganisms in bioaerosols were psychrophilic bacteria and fungi. Bacteria, such as *Staphylococcus* spp. and *Enterococcus* spp*.* which may also include potentially pathogenic speciesas well as *Saccharomyces* spp. were relatively rare. Our analyses showed significant spatial variability in the abundance of airborne microorganisms among the sampling sites. Additionally, there were distinct seasonal patterns in the abundance of the different groups of these microorganisms. As a rule, the maximum densities of bacteria, fungi, and *Saccharomyces* spp. were recorded during the summer, while the minimum occurred in winter. Furthermore, the average abundance of psychrophilic bacteria, mesophilic bacteria, and fungi inbioaerosols was higher in the urban areas compared to the coastal areas. The total number of presumptive *Staphylococcus* spp., *Enterococcus* spp*.*, and *Saccharomyces* spp. was similar in both studied areas.

## Introduction

The atmosphere is widely recognized as a natural habitat for airborne microorganisms originating from both aquatic and terrestrial environments, which create bioaerosols [7, 49, 67]. It is estimated that the global concentration of bioaerosols accounts for 1000 Tg of atmospheric particles each year [39, 69]. Bioaerosols comprise airborne microbes with sizes ranging from 0.001 to 100 µm [54, 63, 75]. These biological particles can consist of different types of organisms, live or dead, such as viruses, bacteria, fungi, algae, protozoa, plant cells, and their metabolic products [13, 25, 66]. Bioaerosols make up approximately 30% of total aerosols and originate from natural sources such as animals, humans, soil, water, forests, agricultural areas, and urban environments [7, 12, 23]. They also come from anthropogenic sources, which include industry, solid and liquid waste management, agriculture, livestock, and food processing [24, 61, 63].

According to Shen and Yao [65], Huang et al., [22], and Zhang et al., [75], bioaerosols are central elements of ecosystems because they play a key role in Earth’s climate change, global mean temperature, and the hydrological cycle. They influence physicochemical processes in the atmosphere by altering the energy balance through the absorption and scattering of solar radiation. Bioaerosols absorb and diffuse solar radiation as well as thermal infrared long-wave radiation, directly affecting regional and global radiative forcing [7, 20, 50]. Bioaerosols can contain up to 100 different species of organisms, mostly saprophytes [35]. The fate of these organisms in the air depends on a combination of various meteorological conditions, mainly temperature, relative humidity, wind speed, direct sunlight radiation, and dust [23, 63, 66]. Another factor that may affect the abundance and diversity of microorganisms in bioaerosols, especially in coastal areas, is the proximity of the marine environment with its high salt concentration. Maki et al., [42] indicate that halotolerant bacteria remain viable and become resistant to the harsh environmental conditions prevailing in the atmosphere. These factors mean that air movement over land and sea surfaces is selective for certain taxa, which can be transported to distant locations, potentially affecting ecosystems as well as public health [43].

Previous studies on bioaerosols are mainly focused on airborne microorganisms in indoor environments, such as hospitals (Bielawska—Drozd et al. [5], [33]), residential and public buildings [7, 26], educational institutions [51, 63], landfills [9, 53] and wastewater treatment plants [32, 44]. However, current microbiological studies on airborne microorganisms in marine urban and coastal regions are limited [25, 37, 45]. According to Huertas et al. [23], the findings from the studies on bioaerosols in marine urban and coastal areas, particularly beaches, have a significant impact on tourism legislation. Most environmental tourism regulations consider water quality standards but often neglect air quality standards related to airborne microorganisms, which are important for protecting the health of tourists.

Many studies [3, 4, 57] show that the town and beach of Ustka are polluted by natural and anthropogenic sources. The main sources of pollution in this area include human activity, the Słupia River, and seabird/shorebird populations. Ustka beach with its surf zone is one of the most picturesque and very popular bathing and sunbathing destinations in Poland. Polish and foreign tourists and local inhabitants intensively visit this beach for recreational purposes, making it particularly crowded during the summer months [52]. This results in an almost tenfold increase in the average number of citizens in the town of Ustka during the summer holidays compared to the rest of the year. According to Astel et al., [4], the regular number of citizens in the town of Ustka is around 16 ths, while during the summer season, it increases to about 120 ths. Another significant source of contamination in the town of Ustka is the Słupia River. This river drains a hydrological basin of about 1623 km^2^ and carries wastewater from urban and agricultural areas along with 200—300 thousand m^3^ year^−1^ of natural and anthropogenic sediments into the sea within the vicinity of the studied beach [73]. Additionally, large populations of foraging and resting swans and gulls observed on this beach significantly contribute to the pollution in the town of Ustka and on its beach. Several colonies of gulls are located along the shore, and their population is rapidly increasing in the Ustka region [3, 57]. The number of birds in the study area may reach even 200—250 thousand [46]. According to Gould and Flechter [19] and Jones and White [28], many seabirds, particularly gulls, can excrete substantial amounts (11.2 to 24.9 g day^−1^) of faeces onto the sand of marine beaches. Seabird excreta represent a significant source of faecal indicator bacteria and potentially opportunistic pathogens in coastal environments. Wright et al., [71] demonstrated that animal fecal inputs at recreational beaches can substantially contribute to the microbial load of sand and nearshore environments. Moreover, birds have been shown to carry antibiotic-resistant strains of staphylococci and enterococci, including MRSA and glycopeptide-resistant enterococci [36], underscoring the potential sanitary relevance of avian-derived contamination. Once deposited in sand, these microorganisms may become resuspended and incorporated into the beach bioaerosol through wind action and human activity, potentially influencing airborne microbial exposure in coastal recreational areas.

Therefore, the studies on bioaerosols in marine urban and coastal recreation areas can provide valuable data to support tourist legislation concerning microbiological air quality. For this reason, the objective of this study was to provide information on the density, diversity, spatial distribution, and seasonal variation of culturable airborne microorganisms in marine urban and coastal zones of recreational marine beach (southern Baltic Sea).

## Material and Methods

### Study Area

**Fig. 1:**
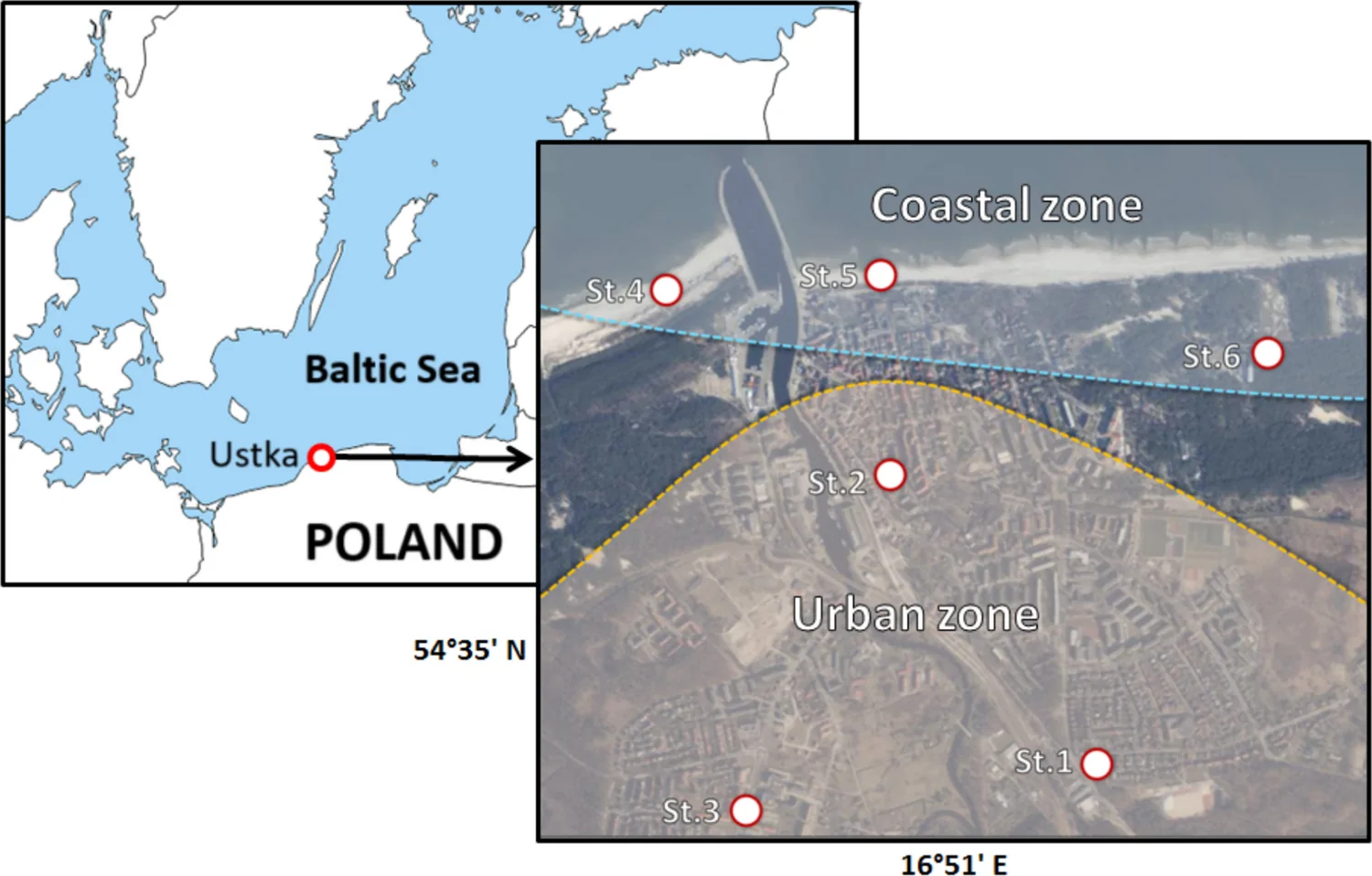
Map of the study area and locations of bioaerosol sampling sites in the marine urban and coastal recreation zone (southern Baltic Sea)

The study area covered the urban coastal zone of a non-tidal sandy beach situated in the town of Ustka (54°35'N 16°51'E) on the southern coast of the Baltic Sea (Fig. 1). It is located at the mouth of the Słupia River, which divides the beach into two parts—the Eastern Beach and the Western Beach. This long beach represents a dissipative type with longshore bars and troughs, with a slope of approximately 7º to 9º and a width ranging from 30 to 55 m [6, 68].

### Air Samples Collection

During the seasonal cycle of 2020, the samples of atmospheric air for microbiological analyses were collected in the town and beach of Ustka from six sites representing urban (st.1,2,3) and coastal (st. 4,5,6) areas (Fig. 1).

st. 1 – located in the suburbs of the town of Ustka,

st. 2 – situated in the town centre (Old City),

st. 3 – situated in a highly urbanized part of the town of Ustka,

st. 4 – located on the Eastern Beach,

st. 5 – located on the Western Beach,

st. 6 – situated in a Spa area with extensive forestation.

**Fig. 2:**
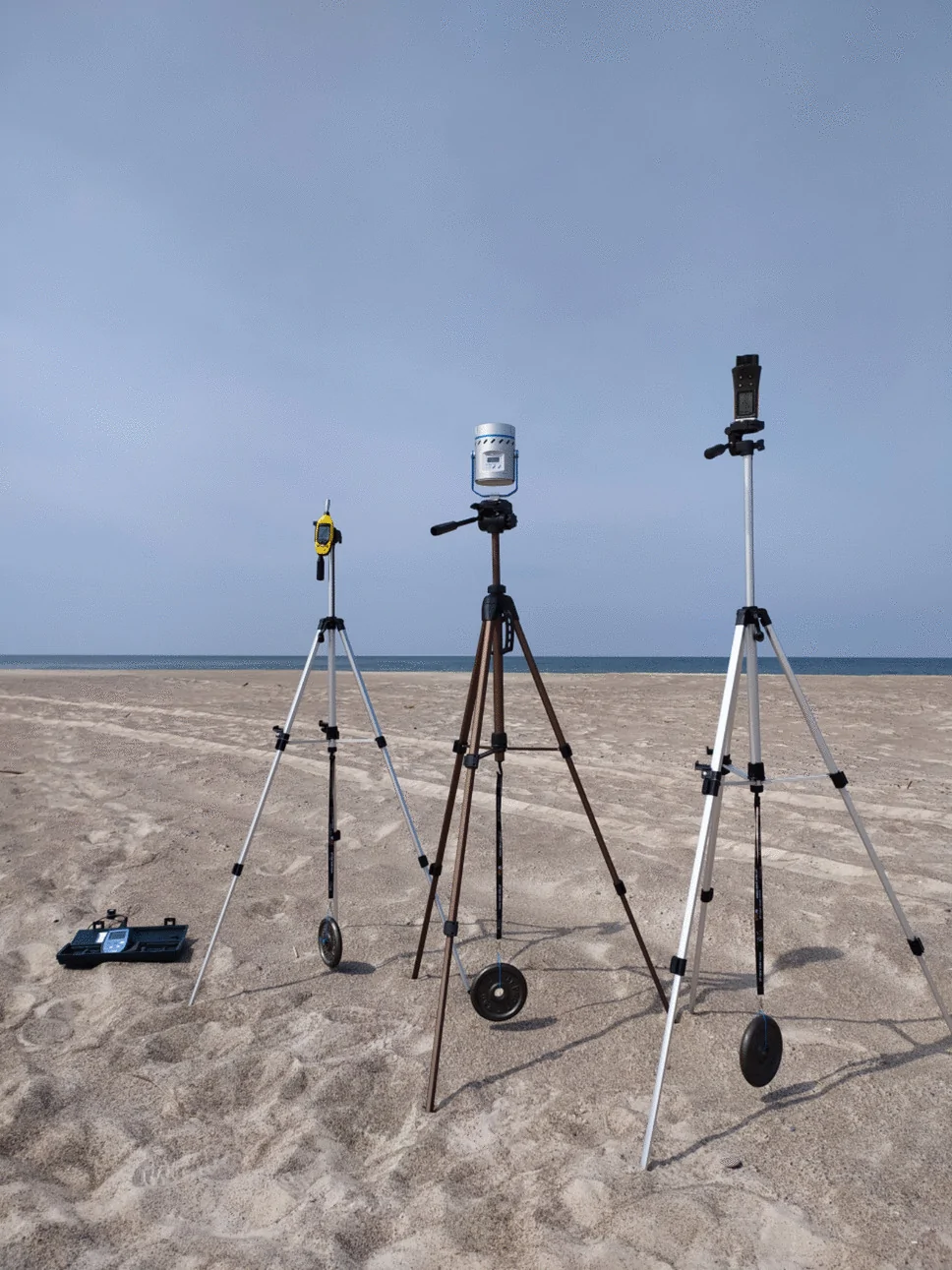
Bioaerosol collections using the MAS-100 Eco air sampler with the impaction method in studied area

Airborne bacteria and fungi were collected using the MAS-100 Eco air sampler (Merck) with the impaction method (Fig. 2). Cut-off diameter (d₅₀) = 1.6 µm and perforated lid 400 × 0.7 mm. Prior to each sampling, the sampler was sterilized with 80% ethanol and set up on a tripod at 1.5 m above ground level, which corresponds to the typical human breathing zone. Sampling was conducted during daylight hours, between 8:00 am and 3:00 pm. A volume of 50–200 L (exposure time 30 s—2 min) of air was filtered in the sampler’s chamber containing Petri dishes (*n* = 72 for each type of medium) filled with a different sterile agar medium (listed below) suitable for the deposition of bacteria, fungi and *Saccharomyces* spp. on their surface. Three replicatesof air samples for each agar medium were always collected at each sampling site. In windy coastal conditions, we using glass Petri dishes, which are not as susceptible to gusts of wind as plastic Petri dishes, which can be blown away by strong winds during field work. After sampling, the agar Petri dishes were immediately placed into a thermostat with ice and transported as soon as possible to the laboratory at the temperature of about 5 °C (transport did not exceed 2-3 h) for further analyses.

### Meteorological Parameters of Air During Sampling

Meteorological parameters, such as air temperature and wind speed, were measured in all sampling sites using a windmeter (Skywatch Meteos). Relative humidity and dust levels were monitored with a dustmeter (BQ20 Trotec) and for solar radiation measurements we used a lightmeter (Delta OHM HD 2302.0) equipped with two probes: one for UVA radiation (Senseca Delta OHM-LP4H UVA, spectral range 315–400 nm), and another for UVB radiation (Senseca Delta OHM-LP4H UVB, spectral range 280–315 nm).

All meteorological parameters were measured parallel with the bioaerosol sample collection. Meteorological data were recorded at the sampling sites and subsequently averaged arithmetically across the sampling period. The meteorological conditions during the sample collection are presented in Table 1.

**Table 1:** Meteorological parameters of the air s during the samples collation

Meteorological parameters	Seasons	Mean	Range	SD
Temperature (°C)	winter	4.5	2.3—6.3	1.36
	spring	11.5	3.3—19.8	6.20
	summer	21.4	16.7—27.0	2.72
	autumn	10.4	1.6—18.6	6.46
Humidity (%)	winter	83.6	75.0—89.0	5.2
	spring	43.6	32.9—53.2	7.2
	summer	52.8	39.0—75.5	9.8
	autumn	73.8	53.7—93.7	13.0
Wind speed (m/s)	winter	5.4	0.3—10.8	3.6
	spring	4.0	0.3—12.0	4.0
	summer	2.2	0.0—7.4	1.8
	autumn	1.0	0.0—3.4	1.0
UVA radiation (W/m^2^)	winter	0.70	0.04—2.22	0.60
	spring	4.70	1.19—11.36	3.14
	summer	5.63	1.71—9.30	2.49
	autumn	1.55	0.14—4.26	1.18
UVB radiation (W/m^2^)	winter	0.015	0.001—0.059	0.02
	spring	0.185	0.022—0.438	0.13
	summer	0.239	0.030—0.460	0.14
	autumn	0.051	0.002—0.179	0.05
PM 2.5 (µg/m^3^)	winter	7.0	4—14	3.3
	spring	5.6	2—9	1.9
	summer	4.2	2—5	0.8
	autumn	7.1	5—13	2.5
PM 10 (µg/m^3^)	winter	11	7—21	3.9
	spring	7.3	3—13	2.6
	summer	5.7	4—7	0.8
	autumn	9.4	5—23	5.2

### Bacteriological and Mycologicalanalyses

All bioaerosols samples were tested to determine the number of microorganisms using traditional culturable methods. According to Wang et al. [70], Huertas et al. [23] and Fan et al. [16], these methods are reliable for assessing the number of microorganisms in bioaerosols. We used suitable selective media to determinethe number of different viable groups of microorganismsin all collected bioaerosols samples. The following media were used:

- psychrophilic bacteria—Tryptone Soya Agar (BTL)—after 7 days of incubation at 20 °C
- mesophilic bacteria—Tryptone Soya Agar (BTL)—after 3 days of incubation at 37 °C
- *Enterococcus* spp.—Slanetz and Bartely LAB-AGAR (Biomaxima S.A)—after 2 days incubation at 37 °C. Faecal enterococcus appears as red or brown colonies
- *Staphylococcus* spp.—Mannitol Salt LAB-AGAR™ (Biomaxima S.A)—after 2 days incubation at 37 °C. Mannnitol-positive and mannitol-negative colonies were counted as *Staphylococcus* spp.
- fungi—Sabouraud Dextrose LAB-AGAR (Merck)—after 7 days of incubation at 25 °C
- *Saccharomyces* spp.—DRBC Agar Base (BTL)—after 5 days of incubation at 25 °C

Each bioaerosols measurement for the tested groups of microorganisms was conducted in triple replications. After incubation, the observable bacterial and fungal colonies were visually enumerated. Their numbers were then corrected using the table of statistical corrections according to Feller [40] and expressed as colony-forming units per cubic meter of air (CFU m^−3^).

### Statistical Analyses

According to Velji and Albright , we calculated several statistical parameters such as standard deviation – SD, standard error – SE, coefficient of variation—CV, and coefficient of dispersion – CD. All statistical analyses were conducted using STATISTICA 13.3. The type of distribution of the variable was assessed using the Shapiro–Wilk normality test. When examining the statistical relationship between two variables, we calculated the correlation coefficient. If the distribution of both variables met the condition of normality, the Pearson linear correlation coefficient was used. If the distribution of at least one variable did not meet the normality condition, then the Spearman rank correlation coefficient was applied. To determine the strength of the relationship between the correlated variables, we used correlogram. The relationships between the studied groups of data were determined based on Spearman’s rank correlation coefficient, calculated in the R environment (version 4.2.1) using the corrplot package. Furthermore, we evaluated the significance of differences in microbiological parameters across the research sites and seasons. For variables meeting the normality condition, we applied ANOVA (analysis of variance) to compare means. If the distribution of the variable did not meet this condition, a nonparametric test was used, i.e., the Kruskal – Wallis ANOVA rank test and the median test.

## Results

The data presented in Table 2 demonstrated that among the culturable microorganisms inhabiting bioaerosols collected from the urban coastal zone of a recreational marine beach, psychrophilic bacteria and fungi were the most abundant. Mesophilic were about three times less numerous than psychrophilic bacteria and fungi. Among other groups of culturable microorganisms inhabiting atmospheric air, a low density was documented in presumptive *Staphylococcus* spp., *Enterococcus* spp., and *Saccharomyces* spp.

**Table 2:** Number microorganisms (colony forming units in 1 m^3^ of atmospheric air—CFU/m^3^) in studied area (average from all sites and seasons)

Microorganism	Statistical parameters				
	Mean (CFU/m^3^)	Range (CFU/m^3^)	SD	CV [%]	CD
psychrophilic bacteria	370	40 – 1663	317	85.6	271.6
mesophilic bacteria	113	0 – 980	165	146.4	241.3
fungi	347	3 – 1213	289	83.2	240.6
*Saccharomyces* spp.	8	0 – 63	12	151.4	18.4
*Staphylococcus* spp.	21	0 – 192	31	149.7	46.6
*Enterococcus* spp.	3	0 – 43	6	276.5	18.1

Explanations:

SDstandard deviation

CV–coefficient of variation

CDcoefficient of dispersion

**Fig. 3:**
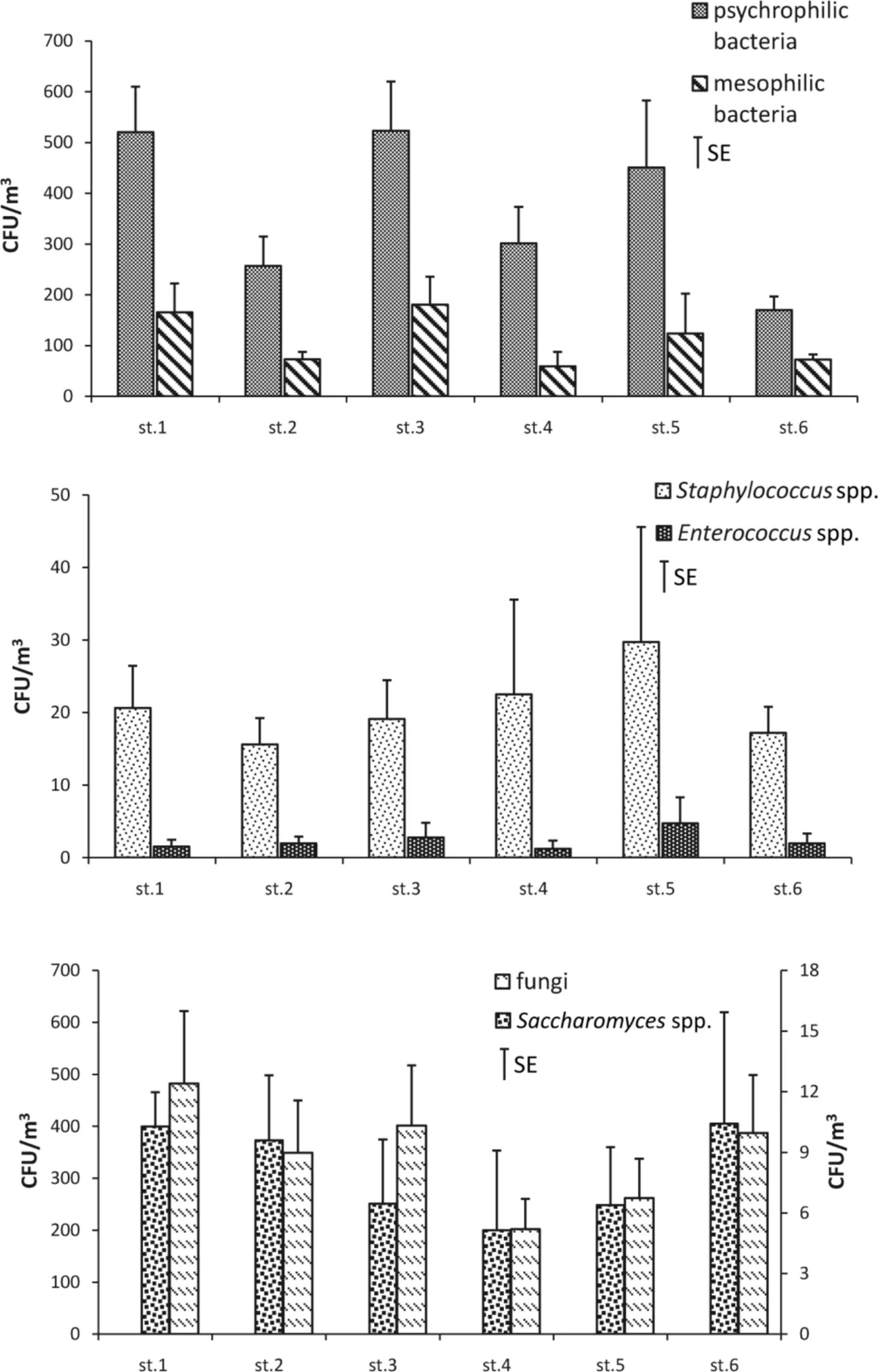
Spatial variability number of culturable airborne microorganisms among different sities studied (average from all seasons). **Abbreviation** CFU/m^3^colony-forming unit per cubic meter of air, SE – standard error

The spatial variability in the number of culturable airborne microorganisms across different sampling sitesis shown in Fig. 3. The highest abundance of psychrophilic and mesophilic bacteria was found at site 1 (the suburbs of Ustka town) and site 3 (a highly urbanized area of Ustka town). The lowest numbers of psychrophilic bacteria were noted at site 6 (a Spa area with extensive forestation) and mesophilic bacteria at site 4 (Western Beach). The highest numbers of presumptive *Staphylococcus* spp. and *Enterococcus* spp. were detected at site 5 (Eastern Beach), whereas the lowest numbers of *Staphylococcus* spp. were observed at site 2 (town centre) and *Enterococcus* spp. at site 4. The highest density of fungi was observed at site 1 and *Saccharomyces* spp.at sites 1, 2, and 3. The lowest numbers of both these groups were found at site 4.

**Fig. 4:**
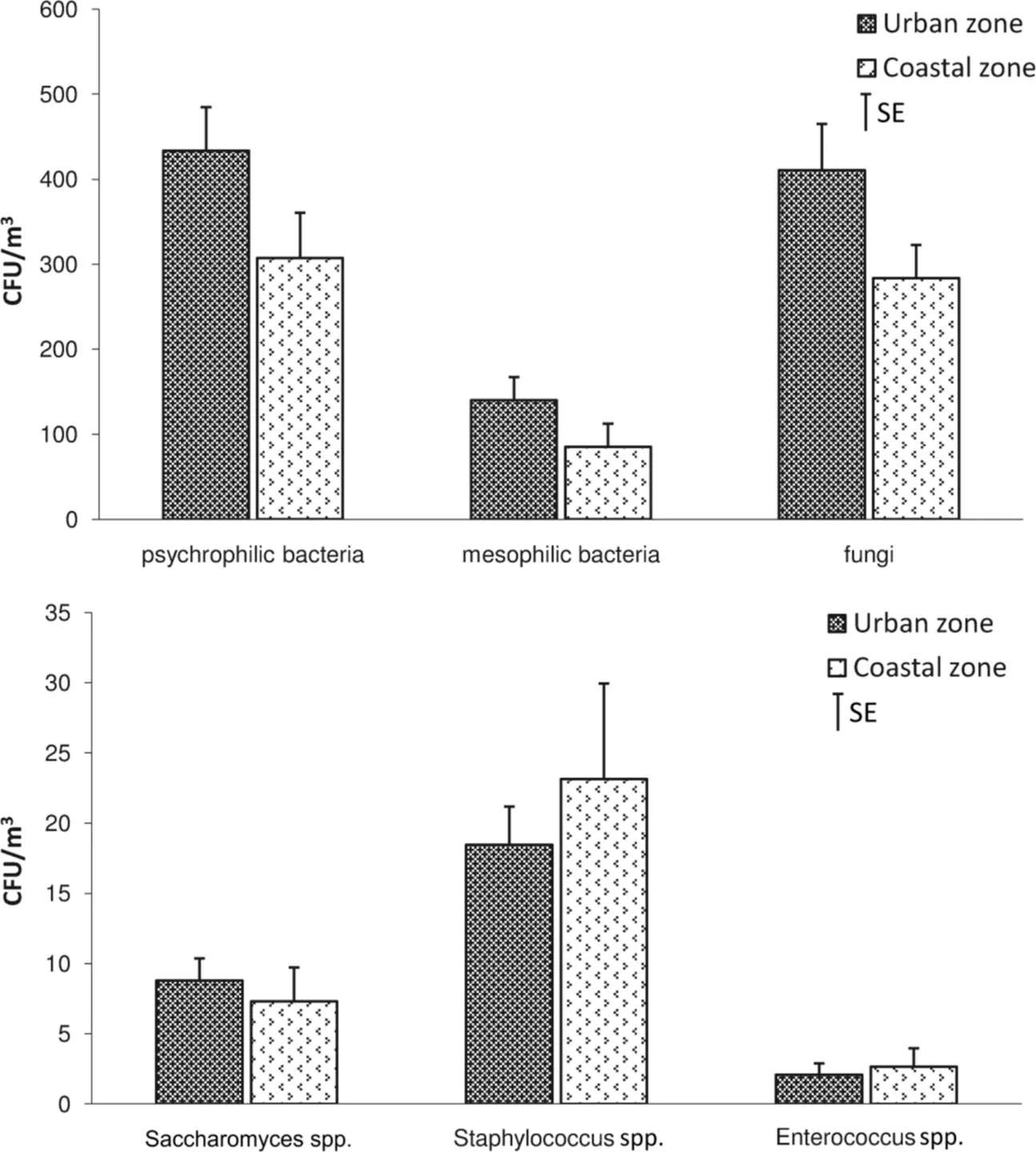
Comparison number of bacteria, fungi and *Saccharomyces* spp. in atmospheric air obtained in the marine urbanized zone and coastal zone (average from all sites and seasons). **Abbreviation** CFU/m^3^colony-forming unit per cubic meter of air, SEstandard error

The numbers of studied microorganisms in bioaerosols differed between urbanized and coastal areas. The mean numbersof psychrophilic and mesophilic bacteria, and fungi were higher in urbanized compared to coastal areas (Fig. 4). However, the presumptive *Staphylococcus* spp. and *Enterococcus* spp*.*, which may also include potentially pathogenic speciesas well as *Saccharomyces* spp. in both studied areas were similar.

Our study documents significant seasonal variation in the abundance of culturable airborne microorganisms (Table 3). Generally, the highest numbers of airborne microorganisms per 1 m^3^ of atmospheric air were noted in summer, while the lowest in winter. During the summer season, the mean levels of mesophilic bacteria, presumptive *Staphylococcus* spp. and *Enterococcus* spp., were approximately nine times higher than in winter. Additionally, the levels of fungi in summer were about five times greater than those in winter.

**Table 3:** Seasonal fluctuation number of airborne bacteria fungi and *Saccharomyces* spp. in studied area (average from all sites)

Microorganism	Seasons	Statistical parameters				
		Mean (CFU/m^3^)	Range (CFU/m^3^)	SD	CV [%]	CD
Psychrophilic bacteria	winter	262	40 – 897	211.0	80.5	169.9
	spring	380	47 – 1267	349.4	92	321.4
	summer	390	60 – 1663	382.5	98	374.8
	autumn	449	37 – 1047	296.2	65.9	195.3
mesophilic bacteria	winter	25	0 – 67	22.2	90.0	19.9
	spring	94	30 – 377	86.4	92.2	79.7
	summer	203	13 – 980	232.5	114.4	266.0
	autumn	129	17 – 660	184.2	142.9	263.4
fungi	winter	135	27 – 473	113.7	84,4	96,0
	spring	167	3 – 470	140.1	83.8	117.4
	summer	613	177 – 1213	283.7	46.3	131.4
	autumn	474	207 – 1107	248.7	52.4	130.0
*Saccharomyces* spp.	winter	3	0 – 15	4.2	124.9	5.2
	spring	6	0 – 23	6.8	116.5	7.9
	summer	17	0 – 63	19.0	112.7	21.4
	autumn	6	0 – 32	9.0	146.1	13.2
*Staphylococcus* spp.	winter	5	0 – 13	4.1	78.0	3.2
	spring	13	0 – 73	17.7	133.9	23.7
	summer	45	13 – 192	50.2	111.4	55.9
	autumn	20	2 – 65	16.1	81.8	13.2
*Enterococcus* spp.	winter	0	0	0	-	-
	spring	0	0	0	-	-
	summer	9	0 – 43	10.8	123.1	13.3
	autumn	1	0 – 5	1.4	218.5	3.1

Explanations:

SDstandard deviation

CV–coefficient of variation

CDcoefficient of dispersion

**Fig. 5:**
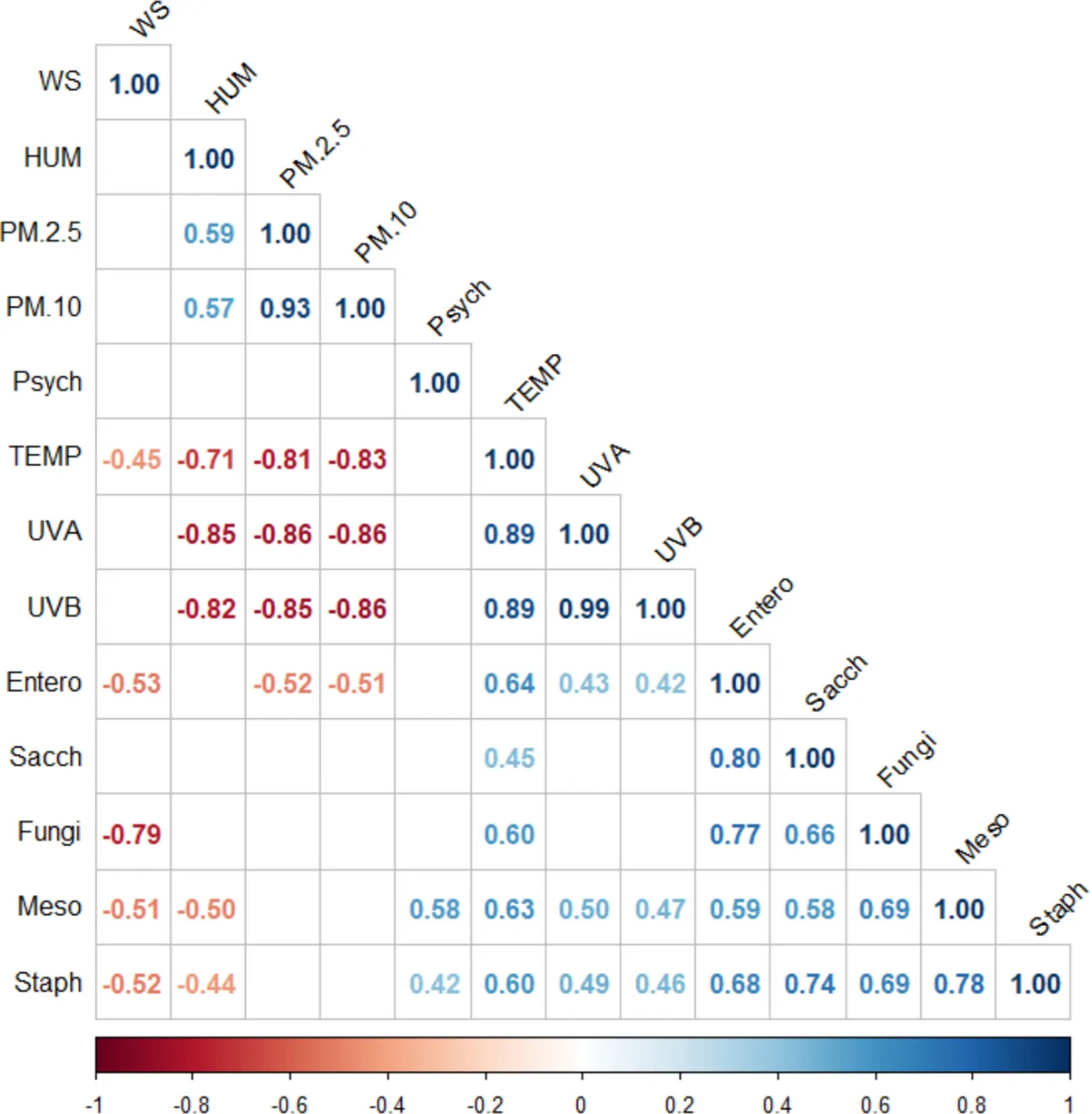
A correlogram illustrating the relationship betweenthe studied groups of data based on Spearman’s rank correlation coefficient (*p* ≤ 0.05). Positive correlations were marked in blue, and negative correlations in red. Color intensity is proportional to the value of the correlation coefficient. White squares indicate correlations that are not statistically significant. **Abbreviations** WSwind speed (m/s), HUMhumidity (%), Psychpsychrophilic bacteria, TEMPtemperature (°C), UVAUVA radiation (W/m^2^), UVBUVB radiation (W/m^2^), Entero*Enterococcus spp*., Sacch*Saccharomyces* spp. Meso – mesophilic bacteria, Staph*Staphylococcus spp*

To analyse the relationships among the studied groups of microorganisms, we conducted a statistical evaluation of the bioaerosols data with *p* ≤ 0.05, which is summarized in the correlogram matrix (Fig. 5). When analysing the abundance of airborne bacteria and fungi inhabiting bioaerosols, we found a very strong positive correlation between the number of mesophilic bacteria and the number of *Staphylococcus* spp. (r = 0.78), as well as fungi (r = 0.69). Moreover, we documenteda strong positive correlation between the density of presumptive *Staphylococcus* spp. and *Enterococcus* spp. (r = 0.68), fungi (r = 0.69), and *Saccharomyces* spp. (r = 0.74).We also observed that the numbers of *Enterococcus* spp. in the studied bioaerosols were significantly positively correlated with fungi (r = 0.77) and *Saccharomyces* spp. (r = 0.80). Furthermore, there was a significant positive correlation between the numbers of fungi and *Saccharomyces* spp. (r = 0.66). The airborne microbiological groups and meteorological conditions revealed a significant positive correlation between temperature and the numbers of mesophilic bacteria (r = 0.63), *Enterococcus* spp. (r = 0.64), and fungi (r = 0.60). Additionally, we found that the number of mesophilic bacteria and *Staphylococcus* spp. showed a positive correlation with both UVA (r = 0.50 and r = 0.49) and UVB radiation (r = 0.47 and r = 0.46). We also documented a negative correlation (r = -0.50) between the number of mesophilic bacteria and humidity, as well as between the number of *Staphylococcus* spp.(r = -0.52), mesophilic bacteria (r = 0.51), fungi (r = -0.79), *Enterococcus* spp. (r = -0.53), and wind speed. Significant negative correlation was also observed between dust PM 2.5 (r = -0.52), dust PM 10 (r = -0.51) and the number of *Enterococcus* spp.

The two-way ANOVA and the Kruskal–Wallis test revealed differences in the number of microorganisms inhabiting bioaerosols grouped by the sites and seasons (Table 4). These analyses showed that the number of mesophilic bacteria *Enterococcus* spp., *Staphylococcus* spp., fungi, and *Saccharomyces* spp. varied significantly between the seasons (*p* ≤ 0.01), but did not differ among the studysites. In contrast, a regular pattern was observed in the number of psychrophilic bacteria.

**Table 4:** Analyses of ANOVA of variance and the Kruskal –Wallis test due to sites sand seasons

Microorganism	Source of variation	H	F	*p*
psychrophilic bacteria	site season		3.043 0.867	* ns
mesophilic bacteria	site season	2.944 12.932		ns **
*Staphylococcus* spp.	site season	1.636 13.938		ns **
*Enterococcus* spp.	site season	0.646 19.272		ns **
fungi	site season	3.509 18.113		ns ***
*Saccharomyces* spp.	site season	5.491 12.972		ns **

Explanations:

FFisher test in ANOVA of variance

Hthe Kruskal – Wallis test

p – significance levelis indicated by asterisks: * *p* ≤ 0.05, ** *p* ≤ 0.01, *** *p* ≤ 0.001

nsnon-significant

## Discussion

Bioaerosols have a biogeochemical association with atmospheric, aquatic, and terrestrial environments, and play a decisive role in the functioning of natural ecosystems and human health [50, 53, 74]. Understanding the atmospheric exchange of viable microbes in aquatic and terrestrial environments is an important yet understudied aspect of microbial ecology and applied environmental microbiology, especially in urban and coastal environments [49]. Measuring the abundance and distribution of culturable bacterial aerosols in coastal urban regions is a practical approach to understanding the transport of viable bacteria originating from both terrestrial and marine sources [61, 69].

The results of the current microbiological study carried out in the urban coastal recreation zone showed that among cultivable airborne bacteria found in bioaerosols, psychrophilic organisms were the most abundant. The density of mesophilic bacteria which are mainly of human and animal origin was approximately three times lower than that of psychrophilic bacteria. These results are consistent with previous research on the number of psychrophiles and mesophiles, and their proportions in bioaerosols over the Gulf of Gdańsk coastal zone (southern Baltic Sea) [35, 45], at health resort [11], and in sewage treatment plants [72]. On the other hand Michalska et al., [47] reported significantly higher numbers of both these groups of bacteria in bioaerosols at Tri-City beaches (Gdańsk-Sopot-Gdynia,southern Baltic Sea), as well as in two regions of Poland [48], and Korzeniewska et al. [31], noted similar results in the atmospheric air around municipal and dairy wastewater treatment plants. Additionally, the results of the present study showed relatively low numbers of presumptive *Staphylococcus* spp. and *Enterococcus* spp. in the analysed bioaerosols. This suggests that the air in the studied area is only slightly polluted with these potentially pathogenic bacteria, which can cause many diseases. The composition and concentration of bioaerosols are influenced by environmental factors, including meteorological conditions, urbanization, and anthropogenic sources [1]. Therefore, monitoring airborne microbial communities is essential for public health protection and the prevention of environmentally related diseases [76]. Our data regarding the number presumptive *Staphylococcus* spp. and *Enterococcus* spp. in bioaerosols fully correspond with the results of studies conducted by Michalska et al., [48] in the air of the Gulf of Gdańsk, Cajazeiras et al., [12] in an Atlantic coastal city, Haas et al. [21] in the air in urban, rural and mountain regions, Breza-Boruta and Paluszak [8] and Małecka-Adamowicz et al., [41] in a wastewater treatment plants.

It should be noted that culturable methods in environmental microbiology have their limitations [32, 55], as only a small proportion of microorganisms can be grown on standard laboratory media, and many cells remain in a VBNC state and do not produce colonies, even though they are alive and metabolically active. For this reason, classical approaches often underestimate the abundance and diversity of microorganisms in environmental samples such as air or water. However, it should be emphasized that studies Prats et al., [60], Drouin and Ferrero [14], Pédron et al., [56] also indicate that well-chosen selective media can effectively isolate part of the environmental microbiota. This means that selective culture still makes sense, especially if we are interested in specific groups of bacteria, and can continue to be a reliable source of useful information about microorganism populations found in various environments. Cultivation methods can be considered as a “first step” in research, which is often justified by the price and availability of equipment.

According to El-Morsy [15], Fröhlich-Nowoisky et al. [18], and Rubiano-Labrador et al. [63] fungi in bioaerosols are abundant and represent about 30% of all atmospheric aerosols. In our study fungi were found to be the second most abundant bioaerosols microorganisms after psychrophilic bacteria. These findings correspond with reports from various studies that have documented the presence of fungi in the air. For instance, Haas et al. [21] studied air samples in Graz, Austria, Jiřík et al. [27] focused on the suburbs of Ostrava, Czech Republik, Hubertas et al. [23] examined a tropical coastal region in Columbia and Ruíz-Fonseca and Rubiano-Labrador [64] investigated urban centres. Fungi most often recorded in bioaerosols include *Alternaria, Cladosporium, Mucor, Ascomycota, Aspergillus*and *Penicillium* [12, 30, 69]*.* Qi et al. [61] also highlight that fungi, mainly those in the 2—5 µm size range, may be transported as spores and fungal hyphae clefts for hundreds of kilometres from their origin and influence the health risk of human populationson a regional scale rather than just locally.

The results of several earlier microbiological research on bioaerosols [25, 29, 49, 53] demonstrated the spatial variability in the distribution of microorganisms among the sampling sites. This spatial variability is probably caused by different meteorological parameters or climatic conditions, and various local natural and anthropogenic sources of bioaerosols emissions at each study site (Breza—Boruta and Paluszak 2007, Qi et al. [61], Rubiano—Labrador et al., 2022). The results of the present study also demonstrated spatial variability in the abundance of culturable airborne microorganisms among the sampling sites. The highest abundance of psychrophilic and mesophilic bacteria, as well as fungi was found at the sites located in the urbanized part of Ustka. The maximum numbers of presumptive *Staphylococcus* spp. and *Enterococcus* spp. were noted in both sites at the beach, where as the lowest numbers of *Staphylococcus* spp. were recorded in the town centre of Ustka. The low numberof these potentially pathogenic bacteria in the Old City of Ustka was probably caused by the fact that during the period of bioaerosols sampling the movement of people in this part of the town was negligible due to the lockdown caused by the COVID-19 pandemic.

The present results showed that the abundance of airborne microorganisms varies significantly between the studied areas. Generally, the average abundance of psychrophilic and mesophilic bacteria, and fungi in bioaerosols was higher in the urban areas compared to the coastal areas. A similar relationship was documented by previous studies [11, 22, 35, 62]. Hurtado et al., [25] reported that in Tijuana, Mexico, the density of microbes in urban air was 200 times higher than in a coastal reference area. The high accumulation of airborne microbes in the urban zone in Ustka may result from the fact that this town is located at the intersection of land and sea. The town is heavily exposed to anthropogenic contamination, moreover, bioaerosols in its urban areas have a special composition due to the accumulation of microorganisms not only of urban terrestrial origin but also those generated by the breaking of marine bubbles and waves [45], Qi et al. [61], Sialve et al. [66], [22].

The results of our study also documented a seasonal variation in the density of airborne bacteria, fungi and *Saccharomyces* spp. Generally, the maximum number of these microorganisms was recorded in summer and minimum in winter. Similar data have been reported by Kuruczalak et al. (2002), Breza-Boruta and Paluszak [8], Fang et al. [17], and Hurtado et al. [25]. The seasonal dynamics in the number of microorganisms in aerosols depends on several meteorological conditions, includingwind direction and speed, relative humidity, dust, solar radiation, and biological aerosol particles [7, 10, 12]. According to Qi et al. [61], Kowalski and Pastuszka [34] and Rubiano—Labrador et al. (2022), the most important environmental factor determining the number of airborne microbes arethermal conditions. The relatively high temperatures in summer directly govern metabolic microbes’ activity and their growth, resulting in their high abundance. This abiotic factor also facilitates the transport of bioaerosols, since the atmospheric water vapour absorbed by these particles increases their weight, and consequently their gravitational deposition [50, 61]. In contrast, the low temperatures in winter may result in poorer survival condition can inhibit the growth of most airborne microbes [37]. Similar results regarding the lowest number of microorganisms in bioaerosols, including psychrophilic bacteria, were recorded in Gdańsk Bay [35].

Lighthart [38] and Hurtado et al., [25], suggest that the relative abundance of Gram-positive cocci during the summer season might result from their structural cell wall composition, which is more resistant to hostile environments such as desiccation andelevated solar radiation levels found in the atmosphere. Furthermore, Amann et al. [2] and Hurtado et al., [25] state that humans inhale approximately 10 m^3^ of air per day. Based on these findings and the data collected in our study, residents of the populations neighbouring the studied areas may inhale around 450 *Staphylococcus* spp., 90 *Enterococcus* spp., and 6300 fungal cells each day. There are currently no international standards for the concentration of bacteria and fungi in outdoor air. In Poland, since 1989, there were standards [58, 59] that specified the measurement methods and permissible concentrations of microorganisms in indoor air. However, these were withdrawn in 2015 and have not been replaced by any other recommendations. We hope that our research indicating potential risks associated with bioaerosols in outdoor air could contribute to and provide arguments for the development of such standards in the future.

In conclusion, we believe that the results of this study documenting the abundance, spatial variability, and seasonal variations of culturable airborne bacteria, fungi and *Saccharomyces* spp. will enrich the limited knowledge about microbiological air quality and potential health risks for residents and tourists staying in urban and coastal areas of recreational marine beaches. We also hope that our findings will be helpful in the potential development of air quality standards, which currently do not exist for such specific areas.

## Data Availability

Data will be made available on request.
